# Untangling biological complexity: A deep learning approach to separating multiple signals in single-cell data

**DOI:** 10.1016/j.xgen.2026.101188

**Published:** 2026-03-11

**Authors:** Christopher Yau

**Affiliations:** 1 Nuffield Department for Women’s & Reproductive Health, University of Oxford, Oxford, UK; 2 Health Data Research UK, London, UK

## Abstract

Single-cell RNA sequencing (scRNA-seq) provides an instantaneous snapshot of the transcriptional state of a cell, which results from the simultaneous activity of many cellular processes. In this issue of *Cell Genomics*, Chen et al.^1^ describe the development of CellUntangler, a deep-learning-based model that allows the capture and filtering of multiple biological signals in scRNA-seq data.

## Teaser

Single-cell RNA sequencing (scRNA-seq) provides an instantaneous snapshot of the transcriptional state of a cell, which results from the simultaneous activity of many cellular processes. In this issue of *Cell Genomics*, Chen et al. describe the development of CellUntangler, a deep-learning-based model that allows the capture and filtering of multiple biological signals in scRNA-seq data.

## Main text

At any instant, a cell harbors a multiplicity of biological activities that govern both its internal regulation and its interactions with other cells. These activities include core housekeeping functions, responses to extracellular cues, and dynamic programs such as differentiation, stress responses, or cell-cycle progression. Single-cell RNA sequencing (scRNA-seq)^2^ provides a quantitative snapshot of the transcripts produced by all of these concurrent processes, offering a window into cellular heterogeneity. In controlled experimental settings, the goal is often to isolate a specific biological process and perturb it—by promoting or inhibiting its activity—in order to observe downstream effects on cellular behavior and phenotype. When the readout is a transcriptional profile, differential expression between a baseline control (wild-type) and a perturbed condition is commonly used to quantify these effects. Such changes are most readily detected when the induced signal dominates background transcriptional activity. However, many biologically meaningful perturbations produce subtle transcriptional effects that are comparable in magnitude to the activity of other ongoing cellular programs. In these cases, the transcriptional signature of interest may be obscured by stronger, unrelated signals. Disentangling overlapping expression patterns therefore represents a fundamental challenge for the analysis and interpretation of scRNA-seq data.

This challenge becomes even more acute when the background processes themselves are of biological interest. Cell-cycle progression provides a canonical example. Because cell-cycle genes are strongly and coordinately regulated, variation in cell-cycle state can dominate scRNA-seq profiles, often eclipsing transcriptional differences associated with cell type, differentiation, or disease. Traditional approaches typically treat such signals as confounders to be computationally removed, for example by regressing out predefined sets of cell-cycle genes prior to downstream analysis.^3^ While effective in certain contexts, this sequential strategy has important limitations. It requires multiple preprocessing steps, relies on assumptions about linearity and independence, and risks removing biologically relevant variation. Most importantly, regression-based approaches discard the very signals they remove, precluding their separate analysis and interpretation.

In this issue, Chen et al.^1^ introduce CellUntangler, a method designed to address this limitation by explicitly separating multiple biological signals within scRNA-seq data rather than collapsing them into a single representation. The approach builds on the framework of variational autoencoders (VAEs), a class of deep generative models that have become increasingly popular in single-cell analysis.^4^ VAEs project high-dimensional gene-expression profiles into probability distributions in lower-dimensional latent spaces using neural networks trained to compress information while preserving the structure of the original data. Through this compression, stochastic gene-expression variability and technical noise are attenuated, while coordinated patterns of gene regulation are retained.

**Figure 1:**
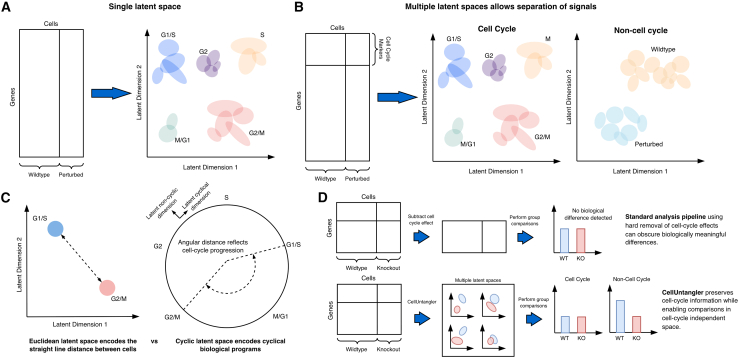
CellUntangler separates overlapping biological signals using decomposed latent spaces with tailored geometries (A) Standard variational autoencoder (VAE) analysis projects all cells into a single latent space, where dominant signals such as cell-cycle progression obscure other biological variation (e.g., differences between wild-type and perturbed conditions). (B) CellUntangler projects cells into multiple latent spaces, each associated with marker genes for a biological process of interest. Cell-cycle variation is captured in one latent space (left), while other biological signals are preserved in a complementary space (right), revealing differences between wildtype and perturbed cells. (C) CellUntangler employs geometrically diverse latent spaces tailored to biological signal structure. Euclidean latent spaces (left) measure straight-line distances between cells, appropriate for discrete cell types or states. Hyperbolic latent spaces (right) use angular distance to represent cyclic processes such as the cell cycle, avoiding artificial boundaries imposed by linear geometries. (D) Top: standard regression-based approaches remove cell-cycle effects before analysis but risk inadvertently removing biologically meaningful variation, potentially masking true differences between experimental groups. Bottom: CellUntangler preserves cell-cycle information in a separate latent space while simultaneously revealing biological differences in a cell-cycle-independent space, enabling parallel interrogation of both signals.

As a result, cells with similar transcriptional programs tend to cluster together in latent space, whereas dissimilar cells are separated. This property has made VAEs powerful tools for denoising, batch correction, and data integration in scRNA-seq studies. However, classical VAE-based approaches typically learn a single latent representation per cell, implicitly encoding all active biological processes into one embedding. When multiple processes operate simultaneously, the dominant signal often defines the global structure of the latent space. In practice, this means that embeddings frequently organize cells according to cell-cycle phase, even when other biological variables are of primary interest (Figure 1A).

CellUntangler departs from this paradigm by learning multiple structured latent spaces, each associated with a predefined set of marker genes corresponding to a biological process of interest (Figure 1B). Rather than forcing all sources of variation into a single representation, the model decomposes the latent space into distinct components, allowing individual signals to be captured separately. For example, by partitioning the input gene set into cell-cycle and non-cell-cycle markers, CellUntangler learns two complementary latent representations: one that captures variation driven by cell-cycle progression and another that focuses on cell-cycle-independent processes. This explicit decomposition allows cell-cycle effects to be isolated rather than removed, enabling downstream analyses to interrogate both cell-cycle dynamics and other transcriptional programs within the same dataset (Figure 1D).

Beyond signal separation, CellUntangler introduces an additional layer of flexibility by allowing each latent space to adopt a geometry appropriate to the biological process it encodes. This design choice reflects the observation that different biological programs impose different structures on gene-expression space. In the case of the cell cycle, progression is inherently periodic, with no natural beginning or end. To accommodate this property, Chen et al. employ a hyperbolic latent space^5^ in which angular distance reflects progression through cell-cycle phases, while radial distance captures additional sources of variation (Figure 1C). This contrasts with standard Euclidean latent spaces, which measure straight-line distances between cells and impose artificial boundaries on cyclic processes. By matching latent-space geometry to biological structure, CellUntangler provides a more faithful representation of cyclic programs such as the cell cycle.

Importantly, this framework is not limited to cell-cycle analysis. Chen et al. demonstrate that alternative gene sets and geometries can be used to disentangle a wide range of overlapping biological and technical signals. One illustrative application involves spatial zonation in liver hepatocytes,^6^ where transcriptional variation reflects both spatial position along the liver lobule and temporal dynamics driven by circadian rhythms. Using a set of spatial marker genes, CellUntangler constructs a latent space that captures the portal-to-central zonation gradient, while a separate latent representation isolates circadian effects. Remarkably, the spatial embedding can be interpreted as a continuous “pseudospace” coordinate, accurately reconstructing known zonation patterns of genes such as Cyp2f2 (portal) and Cyp2e1 (central). By separating spatial and temporal signals, the method enabled identification of a small population of interferon-responsive hepatocytes that had been missed in the original analysis, highlighting how signal disentanglement can reveal rare or subtle cell states.

CellUntangler also proves valuable for addressing technical confounders that frequently obscure biological interpretation. Tissue dissociation during sample preparation is known to induce strong transcriptional stress responses that can dominate scRNA-seq data, particularly in fragile or rare cell types. In an analysis of mast cells from healthy individuals and patients with eosinophilic esophagitis,^7^ Chen et al. use a set of 34 tissue-dissociation marker genes to capture this technical artifact in a dedicated latent space. This separation reveals disease-associated transcriptional differences in a complementary latent representation, allowing the identification of ITGA2B upregulation in active disease—an effect that remained obscured in standard analyses.

A particularly challenging scenario for many single-cell methods involves datasets containing mixtures of cycling and non-cycling cells. Methods that assume all cells follow a shared trajectory often fail in such settings, either by forcing non-cycling cells onto a circular manifold or by clustering cycling cells separately from their true cell-type identities. CellUntangler addresses this challenges by explicitly modeling cycling and non-cycling variation in separate latent spaces. In a large human myeloid cell atlas,^8^ the method places cycling cells along the edge of a hyperbolic disk corresponding to their cell-cycle position, while non-cycling cells cluster near the center. In the complementary latent space, both cycling and non-cycling cells are integrated according to cell type, enabling accurate classification of cycling cells that would otherwise form isolated clusters. This approach led to the reinterpretation of cells previously annotated as doublets, revealing that many corresponded to alveolar macrophages based on marker-gene expression.

The method further demonstrates its versatility in developmental contexts where multiple dynamic processes unfold simultaneously. During mouse pancreatic endocrine development,^9^ both cell-cycle progression and differentiation trajectories shape transcriptional variation. By assigning separate hyperbolic latent spaces to each process, CellUntangler disentangles these overlapping signals, revealing fine-grained developmental progressions that are obscured when both compete for representation in a single embedding. Notably, this analysis resolves distinct trajectories for Acsl1^+^ and Acsl1^−^ epsilon cell precursors, highlighting how disentanglement can sharpen developmental inference.

Finally, Chen et al. show that CellUntangle scales to large datasets, applying the method to nearly one million cells from high-grade serous ovarian cancer samples.^10^ By separating interferon-response signatures from cell-type identity, the analysis reveals that interferon signaling extends across a broader range of cell types in the tumor microenvironment than previously recognized, beyond the lymphocytes highlighted in earlier studies. This result underscores the potential of signal disentanglement to reshape biological conclusions drawn from complex single-cell datasets.

Together, these examples illustrate how CellUntangler reframes a central challenge in single-cell analysis: rather than asking which signal to remove, the method enables multiple signals to be captured, separated, and interrogated in parallel. By combining decomposed latent spaces with biologically informed geometries, CellUntangler provides a flexible framework for untangling overlapping sources of variation in scRNA-seq data. As single-cell studies increasingly probe complex tissues, perturbations, and disease states, approaches that preserve and disentangle biological complexity will be essential for extracting meaningful insight.

## Acknowledgments

The author is supported by an EPSRC Turing AI Acceleration Fellowship (Grant Ref: EP/V023233/1).

## Declaration of interests

The author declares no competing interests.
